# Expression and prognostic significance of PDGF ligands and receptors across soft tissue sarcomas

**DOI:** 10.1016/j.esmoop.2020.100037

**Published:** 2021-01-29

**Authors:** M. Brahmi, T. Lesluyes, A. Dufresne, M. Toulmonde, A. Italiano, O. Mir, A. Le Cesne, T. Valentin, C. Chevreau, S. Bonvalot, N. Penel, J.-M. Coindre, S. Le Guellec, F. Le Loarer, M. Karanian, J.-Y. Blay, F. Chibon

**Affiliations:** 1 Centre Léon Bérard, Lyon, France; 2 University of Bordeaux, Bordeaux, France; 3 Inserm U1218, Institut Bergonié, Bordeaux, France; 4 Inserm UMR1037, Cancer Research Center of Toulouse, Toulouse, France; 5 Institut Claudius Regaud, Toulouse, France; 6 Department of Medical Oncology, Institut Bergonié, Bordeaux, France; 7 Department of Cancer Medicine, Gustave Roussy, Villejuif, France; 8 Department of Medical Oncology, Institut Curie, Paris, France; 9 Department of Medical Oncology, Centre Oscar Lambret, Lille, France; 10 Université Claude Bernard, Lyon, France

**Keywords:** sarcoma, PDGF, PDGFR, expression level, prognostic factor

## Abstract

**Background:**

While the anti-PDGFRA antibody olaratumab failed to confirm an impact on survival in unselected advanced soft tissue sarcoma (STS) patients, the level of expression and the prognosis of platelet-derived growth factor (PDGF) receptors and ligands in STS remain unclear.

**Patients and methods:**

We analyzed PDGF ligands and receptors' expression levels in a series of 255 patients with different histologies of STS [gastrointestinal stromal tumor (GIST), myxoid liposarcoma (MLPS), sarcoma with complex genomics, synovial sarcoma (SyS)] with Agilent single-color micro-arrays. We explored expression levels as prognostic values in univariate and multivariate analysis using R software (version 3.4.2).

**Results:**

Complex patterns of correlation of expression between ligands and receptors were observed for each histotype. PDGFA levels were highest in SyS and lowest in MLPS (*P* < 4 × 10^−9^), PDGFB and C levels were lower in GIST (*P* < 2 × 10^−15^ and *P* < 3 × 10^−9^) while PDGFD expression was similar across histological subtypes. PDGF receptor (PDGFR) A expression was lowest in MLPS (*P* < 0.002), whereas PDGFRB and L expressions were lowest in GIST and SyS (*P* < 0.0004). Interestingly, high PDGFA expression levels were associated with higher risk of metastasis (*P* = 0.006), whereas PDGFD levels above average were associated with a reduced risk of metastasis (*P* = 0.01) in univariate and multivariate analysis.

**Conclusions:**

The expression of PDGF ligands and receptors varies across sarcoma histological subtypes. PDGFA and D expression levels independently and inversely correlate with the risk of metastatic relapse.

## Highlights

• The expression of PDGF ligands and receptors substantially varies across sarcoma histological subtypes.
• PDGFA and D expression levels independently and inversely correlate with the risk of metastatic relapse.
• The differential expression of ligands might be used as biomarker of efficacy for PDGFRα antibodies in STS.

## Introduction

Soft tissue sarcomas (STSs) are rare malignant tumors accounting for <1% of malignant neoplasms.^1^^,^^2^ In most countries, the incidence of STS is estimated at 6 per 100 000 individuals per year with 4000 new cases diagnosed each year in France.^3^ Sarcoma gather a variety of rare histological and molecular subtypes, with >150 distinct subtypes described in the 5th World Health Classification (WHO) Classification of Soft Tissue and Bone Tumors. Management of STS is challenging due to the rarity and the clinical and biological heterogeneity. Standard treatment of advanced/unresectable disease is chemotherapy.^4^ Whereas multiagent chemotherapy with anthracycline plus ifosfamide improves progression-free survival (PFS),^5^ no superiority to single-agent chemotherapy with doxorubicin alone has been evidenced in terms of overall survival (OS). Thus, anthracycline monotherapy providing objective responses in 12%-26% of patients and median PFS of 4-6 months remains the standard first-line treatment.^5^^,^^6^

Platelet-derived growth factor receptor (PDGFR) and its ligand PDGF have been proposed to play an important role in the growth of several cancers.^7^, ^8^, ^9^, ^10^ Genomic alterations of PDGFB and PDGFRA genes are therefore well documented in dermatofibrosarcoma protuberans (DFSP) and subsets of gastrointestinal stromal tumors (GISTs), with translocation and nonsynonymous mutations as key oncogenic events. Not only the discovery of these genetic alterations has been helpful to identify targeted treatments (imatinib), but it is also useful as prognostic and predictive parameters (in particular for GIST).^11^ Besides DFSP and PDGFRA-mutated GIST, the role of PDGFR and the according ligands in the biology of sarcomas remains unclear.

Olaratumab is a fully humanized immunoglobulin G subclass 1 (IgG1) monoclonal antibody that selectively binds to the human PDGFRα, blocking binding and interaction with PDGF and therefore preventing receptor activation.^12^ In a randomized phase II study,^13^ the combination of doxorubicin and olaratumab compared with doxorubicin alone in advanced STS patients significantly improved median OS. While no precise biomarker had been identified, these results suggested that the PDGF–PDGFRα axis plays an important role in the growth of human STS, and a phase III trial which randomized doxorubicin alone versus doxorubicin and olaratumab in advanced or metastatic STS has been conducted in 2016. Unfortunately, the study did not met the primary endpoint of OS^14^ and the combination failed to improve patients outcome.

Following these disappointing results, we managed to investigate the expression and prognostic significance of PDGF ligands and receptors in a retrospective series of STS using microarrays. We hypothesized that not only the expression profiles could highly differ according to the histotype but it could also represent prognostic factors.

## Methods

### Patients

Patients with a diagnosis of STS confirmed by a central histological review based on the French Sarcoma Group (FSG) guidelines for whom tumor samples have been analyzed within the ATGsarc database (http://atg-sarc.sarcomabcb.org/; on-demand access) were included in this observational, retrospective study. ATGsarc database compiles transcriptomic, genomic and clinical information from nearly 1000 patients included in the European sarcoma databases for GIST (ConticaGIST) and STS (Conticabase). The histological diagnosis and grading (for non-GIST sarcoma) were established according to the World Health Organization (WHO) Classification of Tumors and to the *Fédération Nationale des Centres de Lutte Contre le Cancer* (FNCLCC) grading system.^15^ GISTs were classified according to the American Forces Institute of Pathology (AFIP) prognostic group.^16^ Tumor samples were obtained from surgical resection of the primary tumor and consisted of frozen tissues stored at −80°C.

### Gene expression studies

The transcription profiles were obtained using Agilent single-color microarrays (model 014850; Agilent, Paris, France). Quantile normalization was performed to normalize expression data. For each gene, we selected the probe that maximizes the interquartile range value (higher expression dispersion) to reflect gene expression. Selected probes (maximum interquartile range) were A_23_P113701 (PDGFA), A_24_P339944 (PDGFB), A_24_P163168 (PDGFC), A_24_P124349 (PDGFD), A_23_P332536 (PDGFRA), A_23_P421401 (PDGFRB) and A_23_P60146 (PDGFRL). Data are available online in the ATGsarc database (http://atg-sarc.sarcomabcb.org/; on-demand access).

### Statistics

Metastasis-free survival, local relapse-free survival and OS were evaluated according to the Kaplan–Meier estimator from the date of the initial diagnosis to the date of the occurrence of the respectively reported event (metastatic relapse, local relapse or death) or the latest follow-up. Significances are given by log-rank tests, where *P* < 0.05 was considered as a significant survival difference between risk groups. Hazard ratios were given by Cox regressions for univariate analyses and Cox regression with Firth's penalized likelihood for multivariate analyses. The following clinical information was considered for survival analyses: grading system, PDGF/PDGFR expression levels (as discrete variables: above and below mean expression), histological subtype, tumor size (as discrete variable: <50 mm and ≥50 mm) and location (non-GISTs). These statistics were performed using R software (version 3.4.2) with survival (version 2.41.3), coxphf (version 1.12) and Rtsne (version 0.13, perplexity value set to 30) packages. The expression levels in the different histological subtypes were investigated using pairwise *t*-tests with Benjamini–Hochberg adjustments. The t-distributed stochastic neighbor embedding (t-SNE) technique was applied for dimensionality reduction as an unsupervised method to identify samples with similar transcriptomic patterns in all cohorts and three dimensions were kept to display 3D scatterplots.

## Results

### Patients

Patients' characteristics are presented in Table 1. The mean age at diagnosis was 50 years (range 1-92) and 59% of patients (*n* = 150/255) were males. The main histologies were GIST (*n* = 60), myxoid liposarcoma (MLPS; *n* = 50), synovial sarcoma (SyS; *n* = 58) and sarcoma with complex genomics (SCG; *n* = 87). The group of SCG gathered undifferentiated pleomorphic sarcomas (*n* = 30), leiomyosarcomas (*n* = 24), dedifferentiated liposarcomas (*n* = 11) and rarer subtypes (*n* = 22) including six myxofibrosarcomas, six pleomorphic rhabdomyosarcomas and four pleomorphic liposarcomas.

**Table 1:** Baseline characteristics of the retrospective series of 255 STS patients

	GIST	MLPS	SyS	SCG	All histotypes
*N* (%)	60 (23.5)	50 (19.5)	58 (23)	87 (34)	255 (100)
Male	34 (57)	32 (64)	33 (57)	51 (59)	150 (59)
Female	26 (43)	18 (36)	25 (43)	36 (41)	105 (41)
Median (range) age (years)	63 (36-76)	44 (16-83)	20 (1-71)	63 (16-92)	50 (1-92)
Median (range) size (mm)	55 (15-280)	110 (40-270)	70 (27-240)	100 (20-300)	80 (15-300)
Site, *n* (%)					
Extremities	0 (0)	46 (92)	41 (71)	61 (70)	148 (58)
Head and neck	0 (0)	0 (0)	5 (8)	0 (0)	5 (2)
Trunk wall	0 (0)	3 (6)	12 (21)	15 (17)	30 (12)
Internal trunk	60 (100)	1 (2)	0 (0)	11 (13)	72 (28)
Grade (FNCLCC/AFIP), *n* (%)					
I/low and very low	29 (48.3)	27 (54)	0 (0)	0 (0)	56 (22)
II/intermediate	14 (23.3)	21 (42)	7 (12)	25 (29)	67 (26)
III/high	17 (28.3)	2 (4)	51 (88)	62 (71)	132 (52)

AFIP, American Forces Institute of Pathology; FNCLCC, *Fédération Nationale des Centres de Lutte Contre le Cancer*; GIST, gastrointestinal stromal tumor; MLPS, myxoid liposarcoma; SCG, sarcoma with complex genomics; SyS, synovial sarcoma.

### Expression levels of PDGF ligands and receptors in sarcoma histotypes

**Figure 1:**
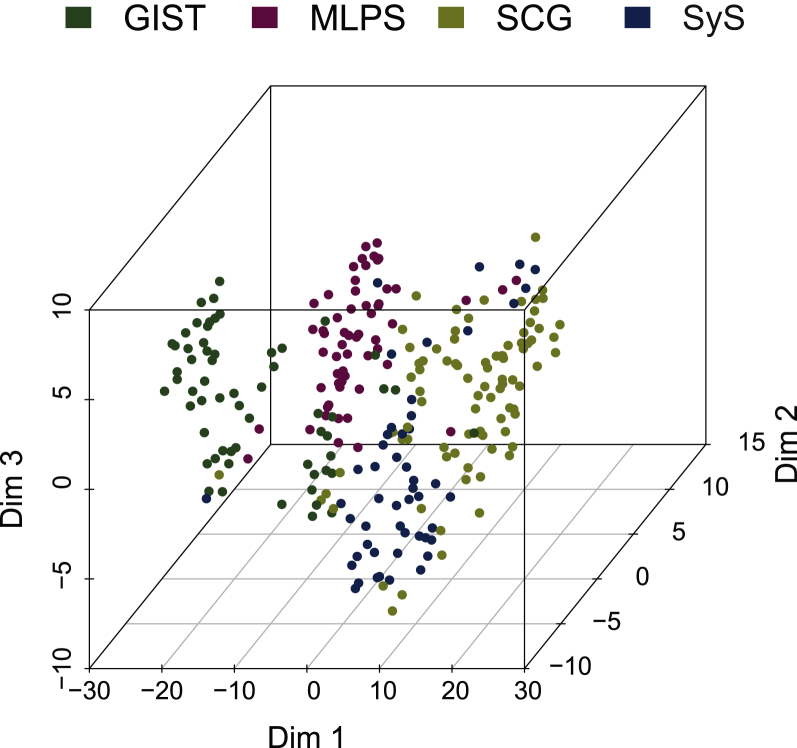
t-SNE (t-distributed stochastic neighbor embedding) analysis on PDGF ligand and receptor expressions in the four sarcoma cohorts. In agreement with differential gene expression, this 3D clustering efficiently discriminated the four different sarcoma subtypes confirming that histological subtypes harbor different expression patterns of ligands and receptors of the PDGF pathway. GIST, gastrointestinal stromal tumor; MLPS, myxoid liposarcoma; PDGF, platelet-derived growth factor; SCG, sarcoma with complex genomics; SyS, synovial sarcoma.

**Figure 2:**
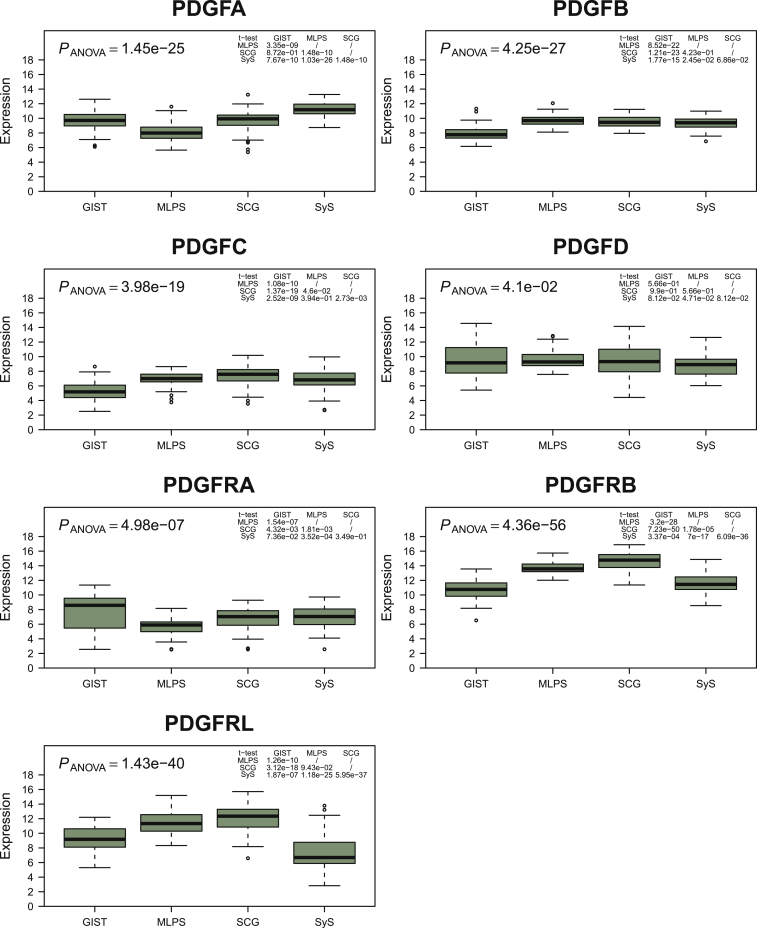
Expression levels of PDGF ligands and receptors according to histotypes. The boxplots show the variation of expression of PDGF ligands and receptors across sarcoma histological subtypes. GIST, gastrointestinal stromal tumor; MLPS, myxoid liposarcoma; PDGF, platelet-derived growth factor; SCG, sarcoma with complex genomics; SyS, synovial sarcoma.

Complex patterns of correlation of expression between ligands and receptors were observed in each of the four subtypes of the series of 255 sarcomas, highlighted by a technique of t-SNE algorithm (Figure 1). This method processes a dimensional reduction to better visualize data. All seven genes (PDGFA/B/C/D and PDGFRA/B/L) contribute to this profile and the three dimensions are informative at similar levels. The four sarcoma histotypes segregated significantly, showing that the different histological subtypes harbor wide different expression patterns. PDGFA levels were highest in SyS and lowest in MLPS (*P* < 4 × 10^−9^), PDGFB and C levels were lower in GIST (*P* < 2 × 10^−15^ and *P* < 3 × 10^−9^), while PDGFD expression was similar across histological subtypes (Figure 2). PDGFRA expression was lowest in MLPS (*P* < 0.002), while PDGFRB and L expression levels were lowest in GIST and SyS (*P* < 0.0004). We also tested the relation between the expression levels of the ligands and receptors and the grade, the size and the site of the primary tumor. In SCG, the primary tumor site was correlated with expression levels of PDGFB and PDGFRB (*P* < 0.05). In GIST, the AFIP score was correlated with expression levels of PDGFRL (*P* = 0.04).

### Prognostic value of expression levels of PDGF ligands and receptors

**Figure 3:**
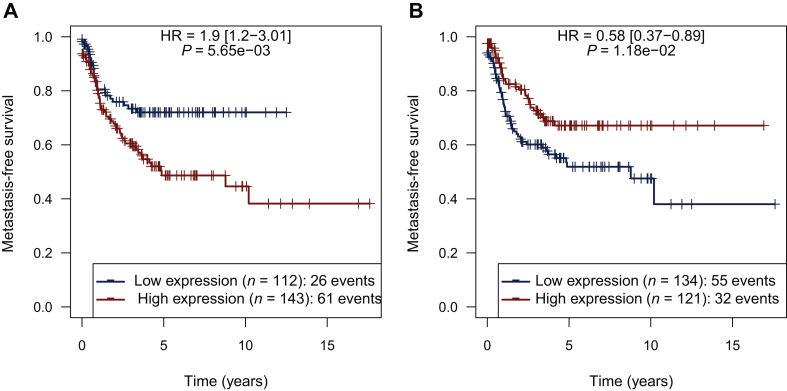
Correlation between expression levels of PDGFA/D and MFS/RFS. (A) PDGFA expression levels above the mean were associated with a higher risk of metastasis. Conversely, (B) PDGFD above the mean was associated with a reduced risk of metastasis. HR, hazard ratio; MFS, metastasis-free survival. PDGF, platelet-derived growth factor; RFS, recurrence-free survival.

We then compared the impact of high expression levels of receptors and ligands on the risk of local relapse-free survival, metastatic relapse survival and death (OS), comparing patients in whom expression levels in sarcoma were above versus under the mean. In the whole cohort, PDGFA expression levels above the mean were associated with a higher risk of metastasis (*P* = 0.006) (Figure 3A), while no differences were observed for PDGFB and PDGFC (*P* = 0.67 and 0.84, respectively). Conversely, PDGFD above the mean was associated with a reduced risk of metastasis (*P* = 0.01; Figure 3B). We did not identify correlations between PDGFRA, PDGFRB and PDGFRL expression levels and relapse (*P* > 0.05). No correlation between expression levels of PDGF receptors and ligands and OS was evidenced.

### Multivariate analysis

In the non-GIST sarcoma cohort, histological subtype, location, grade, tumor size, PDGFA and PDGFD expression levels were introduced in the multivariate model. Tumor size and PDGFA expression levels were identified as independent adverse prognostic factors (*P* = 1.3 × 10^−4^ and *P* = 4.3 × 10^−2^, respectively) while PDGFD expression levels were a favorable prognostic factor for metastasis-free survival (*P* = 4.99 × 10^−3^). Histological subtype, location and grade were not a significant factor in this analysis (*P* > 0.07, *P* > 0.4 and *P* = 0.5, respectively; Table 2). In the GIST cohort, AFIP score, PDGFA and D expression levels were introduced in the multivariate model. The PDGFD expression level was also an independent favorable prognostic factor for metastasis-free survival, in addition to AFIP score (respectively *P* = 3.7 × 10^−2^ and *P* = 9.3 × 10^−5^), while PDGFA was not identified as a significant factor in this analysis (*P* = 0.9).

**Table 2:** Impact of the histological subtype, location, grade, tumor size, PDGFA and D expression levels on MFS in multivariate analysis in the non-GIST sarcoma cohort and impact of the AFIP score, PDGFA and D expression levels on MFS in multivariate analysis in the GIST cohort

	Non-GIST cohort		GIST cohort	
	HR (95% CI)	*P*	HR (95% CI)	*P*
Histological subtype	N/A	>0.07	N/A	N/A
Location	N/A	>0.4	N/A	N/A
Grade	HR 0.8 (0.44-1.52)	0.5	N/A	N/A
Tumor size	HR 5.37 (2.07-19.69)	1.3 × 10^−4^	N/A	N/A
AFIP score	N/A	N/A	HR 26.38 (3.5 to >100)	9.3 × 10^−5^
PDGFA	HR 1.27 (1.01-1.6)	4.3 × 10^−2^	HR 0.98 (0.73-1.38)	0.9
PDGFD	HR 0.8 (0.69-0.94)	4.99 × 10^−3^	HR 0.75 (0.56-0.98)	3.7 × 10^−2^

AFIP, American Forces Institute of Pathology; GIST, gastrointestinal stromal tumor; HR, hazard ratio; MFS, metastasis-free survival; PDGF, platelet-derived growth factor.

## Discussion

This work reports on the differential expression of PDGF ligands and receptors in a series of 255 patients with STS including four different histotypes. The expression of PDGF ligands and receptors substantially varies across sarcoma histological subtypes. In addition, the expression levels of PDGFA and D were observed to correlate independently and inversely to the risk of metastatic relapse. Of note, in the multivariate analysis, the grade was not a significant factor. We hypothesized here that the majority of the SCG and SyS were high-grade tumors and in the MLPS, the percentage of the round cells component is more significant.

The PDGF family of ligands is involved in a variety of biological processes. PDGF modulates the growth, survival and cell function in several types of connective tissue cell.^17^ In humans, PDGF oncogenic role has been reported in several cancers^7^, ^8^, ^9^, ^10^ such as gastric adenocarcinomas,^8^ gliomas,^9^^,^^18^ medulloblastomas,^19^ DFSP and a subset of GIST, suggesting that it contributes to oncogenesis in these models and therefore may constitute a promising therapeutic target.^10^ In human gastric cancers, high levels of PDGFA correlate with high-grade carcinomas and reduced patient survival.^20^ The study here focused on the expression levels of the PDGF ligands and receptors in four molecular subtypes of sarcomas and had not been conducted before to our knowledge. Copy number variations of the seven genes were observed in <5% of the tumors (with occasional losses or gains, and no amplifications). The variations of the expression of the four ligands and the three receptors across the different histotypes and within each single histotypes are thus related to epigenetic events, for which the exact nature and mechanism remain to be characterized. The overexpression of PDGFA in SyS, and the lowest levels observed in MLPS might suggest that the product of the fusion genes might be involved in transcriptional regulation of this gene.

Olaratumab has been tested in a phase II trial with previously treated metastatic or unresectable GIST.^21^ While there was no apparent effect on PFS in patients without PDGFRα mutations, PDGFRα-mutant GIST patients (all with D842V mutations) treated with olaratumab had longer disease control compared with historical data for this genotype. Olaratumab has not yet been tested in DFSP, which bears a translocation involving PDGFA as a nosological hallmark.^13^ Beyond the experience in GIST and DFSP, the network of interactions of PDGF ligands and receptors is complex and the pattern of expression of the PDGF ligands and receptor has not been explored so far as prognostic or predictive marker in the different histological and molecular subtypes of human sarcomas. Despite promising results of the phase I and randomized phase II trial, the combination of olaratumab with doxorubicin in patients with advanced STS failed to achieved a highly significant improvement of outcome in the recent phase III trial ‘ANNOUNCE’.^13^ In the era of targeted therapies, uncovering biomarkers of activity and improving knowledge in mechanisms of resistance are crucial to overcome the current challenges.

The results presented here show that the overexpression of PDGFA might correlate with tumor aggressiveness in sarcoma: overexpression of PDGFA, a PDGFRα-specific ligand, was an independent adverse prognostic factor. These findings, although exploratory, would be interesting to correlate with the clinical results of olaratumab. Indeed, the expression of the ligand might be related to the antitumor activity of a PDGFRA antibody targeting its receptor and these results might warrant further evaluation of olaratumab in SyS. Conversely, PDGF-D was found to be an independent favorable prognostic factor for metastasis-free survival. This fourth member of the ‘PDGF family’ has been identified and characterized as a specific agonistic ligand for PDGFRβ. Indeed, PDGFD exerts its cellular effects by exclusively binding to PDGFRβ. The biological mechanism underlying the favorable prognostic value of PDGFD remains unclear. It should also be remembered that PDGFRL, which is also a specific agonistic ligand for PDGFRβ, is known to have potential tumor suppressor activity. The two receptors alpha and beta activate common signaling pathways [such as cyclic adenosine monophosphate (cAMP)/Ca^+^ signaling, the G-protein-coupled receptor signaling and the phosphoinositide 3-kinase (PI3K) pathway], but some signaling pathways are exclusively or predominantly activated by one receptor but not by the other. PDGFRα/β activates components of the nuclear factor-kappaB and interleukin-6 signaling pathways, PDGFRα activates C21-steroid hormone biosynthesis and PDGFRβ activates the angiogenesis and epidermal growth factor receptor signaling pathways. These two receptors have therefore distinct roles *in vivo*,^22^ with PDGFRβ being implicated in angiogenesis, whereas PDGFRα is implicated in embryogenesis (development of central nervous system, neural crest and organs).^23^, ^24^, ^25^, ^26^ It could be speculated that the activation of the PDGFRα may lead to tumor growth, whereas the activation of PDGFRβ may lead to a tumor suppressor activity.

As a limitation of our study, it is important to mention that it did not include any validation cohort. Thus, future studies of larger cohorts of STS are needed to confirm and to expand on our results, and are currently ongoing in our center, using RNA-seq technology. Moreover, the present results, based on a compared analysis of the messenger RNA expression levels of PDGF ligands and their receptors, are not appropriate to identify a biomarker for the efficacy of a treatment. However, they show that different sarcomas have different expression patterns of these ligands and receptors, and that two different ligands are associated with opposite prognosis significance for the risk of metastatic relapse, including in multivariate analysis. It would be important to further explore the potential predictive value of the expression of these two ligands on the PFS of patients treated with olaratumab.

Overall, this study describes for the first time the expression and prognostic significance of PDGF ligands (A, B, C and D) and receptors (A, B and L) in a large cohort of different subtypes of soft-tissue sarcomas. It shows that expression of these ligands and receptors correlate with sarcoma patient outcomes and indicates that differential expression of the ligands may constitute relevant biomarkers of efficacy of PDGFRα antibodies in this heterogeneous family of diseases.
